# Asymptomatic Cattle Naturally Infected with *Mycobacterium bovis* Present Exacerbated Tissue Pathology and Bacterial Dissemination

**DOI:** 10.1371/journal.pone.0053884

**Published:** 2013-01-09

**Authors:** Álvaro Menin, Renata Fleith, Carolina Reck, Mariel Marlow, Paula Fernandes, Célso Pilati, André Báfica

**Affiliations:** 1 Laboratory of Immunobiology, Universidade Federal de Santa Catarina, Florianóspolis, Santa Catarina, Brazil; 2 Laboratory of Histology and Immunohistochemistry, Universidade do Estado de Santa Catarina, Lages, Santa Catarina, Brazil; 3 Laboratory of Protozoology, Universidade Federal de Santa Catarina, Florianóspolis, Santa Catarina, Brazil; Fundació Institut d’Investigació en Ciències de la Salut Germans Trias i Pujol, Universitat Autònoma de Barcelona, CIBERES, Spain

## Abstract

Rational discovery of novel immunodiagnostic and vaccine candidate antigens to control bovine tuberculosis (bTB) requires knowledge of disease immunopathogenesis. However, there remains a paucity of information on the *Mycobacterium bovis*-host immune interactions during the natural infection. Analysis of 247 naturally PPD+ *M. bovis*-infected cattle revealed that 92% (n = 228) of these animals were found to display no clinical signs, but presented severe as well as disseminated bTB-lesions at *post-mortem* examination. Moreover, dissemination of bTB-lesions positively correlated with both pathology severity score (Spearman r = 0.48; p<0.0001) and viable tissue bacterial loads (Spearman r = 0.58; p = 0.0001). Additionally, granuloma encapsulation negatively correlated with *M. bovis* growth as well as pathology severity, suggesting that encapsulation is an effective mechanism to control bacterial proliferation during natural infection. Moreover, multinucleated giant cell numbers were found to negatively correlate with bacterial counts (Spearman r = 0.25; p = 0.03) in lung granulomas. In contrast, neutrophil numbers in the granuloma were associated with increased *M. bovis* proliferation (Spearman r = 0.27; p = 0.021). Together, our findings suggest that encapsulation and multinucleated giant cells control *M. bovis* viability, whereas neutrophils may serve as a cellular biomarker of bacterial proliferation during natural infection. These data integrate host granuloma responses with mycobacterial dissemination and could provide useful immunopathological-based biomarkers of disease severity in natural infection with *M. bovis*, an important cattle pathogen.

## Introduction

Bovine tuberculosis (bTB), caused by infection with the intracellular acid-fast bacilli *Mycobacterium bovis*, is an important neglected zoonosis, which significantly decreases livestock production and economically affects international trade [1]–[3]. Additionally, *M. bovis* infection is estimated to be responsible for ∼10% of human tuberculosis (TB) in Africa [3] and ∼2.5% of human cases in Latin America [4], thus underscoring the importance of disease control programs based on the understanding of infection dynamics [5]–[8].

Currently, no effective vaccine exists for bovine TB. The main procedures to control/eradicate this intractable disease are diagnosis and compulsory slaughter of positive animals [9]. In this context, the most utilized diagnostic tool for *M. bovis* infection in cattle is the single intradermal comparative cervical tuberculin test (SICTT), which measures a delayed type hypersensitivity response to the tuberculin antigen-purified protein derivative (PPD) [10], but may fail to detect specific pathogen infection [11]–[13]. Indeed, Claridge et al. have recently reported that *Fasciola hepatica* co-infection in bTB diseased cattle significantly decreases the numbers of PPD-positive animals [11], demonstrating PPD sensitivity could be affected by parasitic co-infections. Together, these data indicate an urgent need for an effective vaccine as well as better diagnostic tests to control bTB. However, there remains a paucity of information on bTB immunopathogenesis, especially during natural infection.

*M. bovis* primarily infects macrophages, where they can survive, replicate and disseminate into different anatomical sites [14], [15]. The risk of transmission as well as the host’s survival relies mainly on the ability of well-organized structures called granulomas to contain mycobacterial infection [14]–[17]. Tuberculous granuloma is a complex host-protective structure generated in response to persistent mycobacterial stimuli with focal accumulation of inflammatory cells, such as multinucleated giant cells and lymphocytes [16], [18]–[21]. In addition, encapsulation, a process involving production of connective tissue around the granuloma, has been shown to be critical for controlling both mycobacterial growth and tissue dissemination [14], [15], [22].

The pathological outcome of experimental *M. bovis* infection has been associated with diversity and efficiency of host immune response as well as a useful tool for evaluating efficiency of new vaccine antigen candidates and disease severity [23]–[26]. Furthermore, the presence of cellular populations, such as epithelioid cells, multinucleated giant cells, lymphocytes and neutrophils in the tuberculous granuloma [19], [21] during experimental *M. bovis* infection, suggests these cells may play important roles in controlling bTB. Although genetic variability and age-associated factors have been shown to potentially be involved in susceptibility to *M. bovis* infection [2], [27]–[32], fundamental host defense aspects of the natural infection by *M. bovis* have not been fully elucidated. Consistently, cellular immune responses against this major bovine pathogen as well as the tuberculous granulomatous response elicited during natural infection in cattle are poorly understood.

In the present study, we have performed a detailed analysis of several host immune and pathology response parameters in a cohort of 247 naturally *M. bovis*-infected cattle. Our findings reveal that, despite the absence of clinical symptoms, naturally-infected bovines displayed severe lung pathology and bacterial dissemination, which correlate with viable mycobacterial loads within the granuloma. Furthermore, immune-related cells and tissue remodeling of the granuloma were found to correlate with bacterial containment during natural infection. Our results provide useful insights on possible biomarkers of disease severity in natural infection with an important cattle pathogen.

## Results

### Viable Bacterial Loads Correlate with Tissue Pathology in Naturally *M. bovis-*infected Cattle

**Figure 1:**
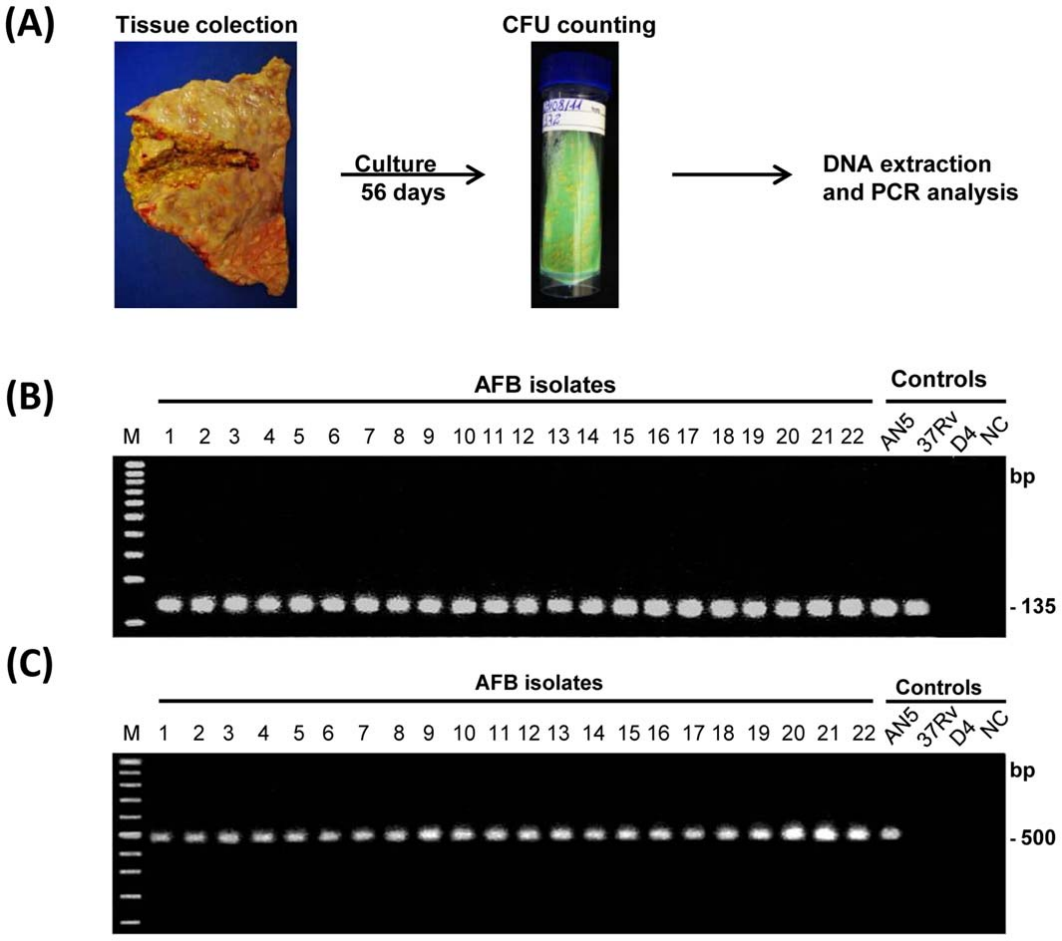
Bacteriology analysis and molecular typing of *M. bovis*. (**A**) Tissue homogenate obtained from PPD+ asymptomatic animals was inoculated in Ogawa-Kudoh+sodium pyruvate and incubated at 37°C for 8 weeks. After that, colonies were counted and DNA extraction method employed. Purified DNA obtained from (**A**) was used as template for PCR amplification of (**B**) IS1081 (∼135 bp) or (**C**) RvD1Rv2031c (∼500 bp) gene sequences. Amplification products of single PCR from representative samples are shown. Lane M: 100 bp DNA ladder; lanes 1–22 PCR products of *M. bovis* isolates; AN5 - *Mycobacterium bovis* AN5 strain, standard strain positive control; 37Rv – *Mycobacterium tuberculosis* strain, H37Rv; D4 - *Mycobacterium avium Subsp. avium D4* strain, non-tuberculosis mycobacteria (NMTBC) member; lane NC, negative control (without DNA).

**Figure 2:**
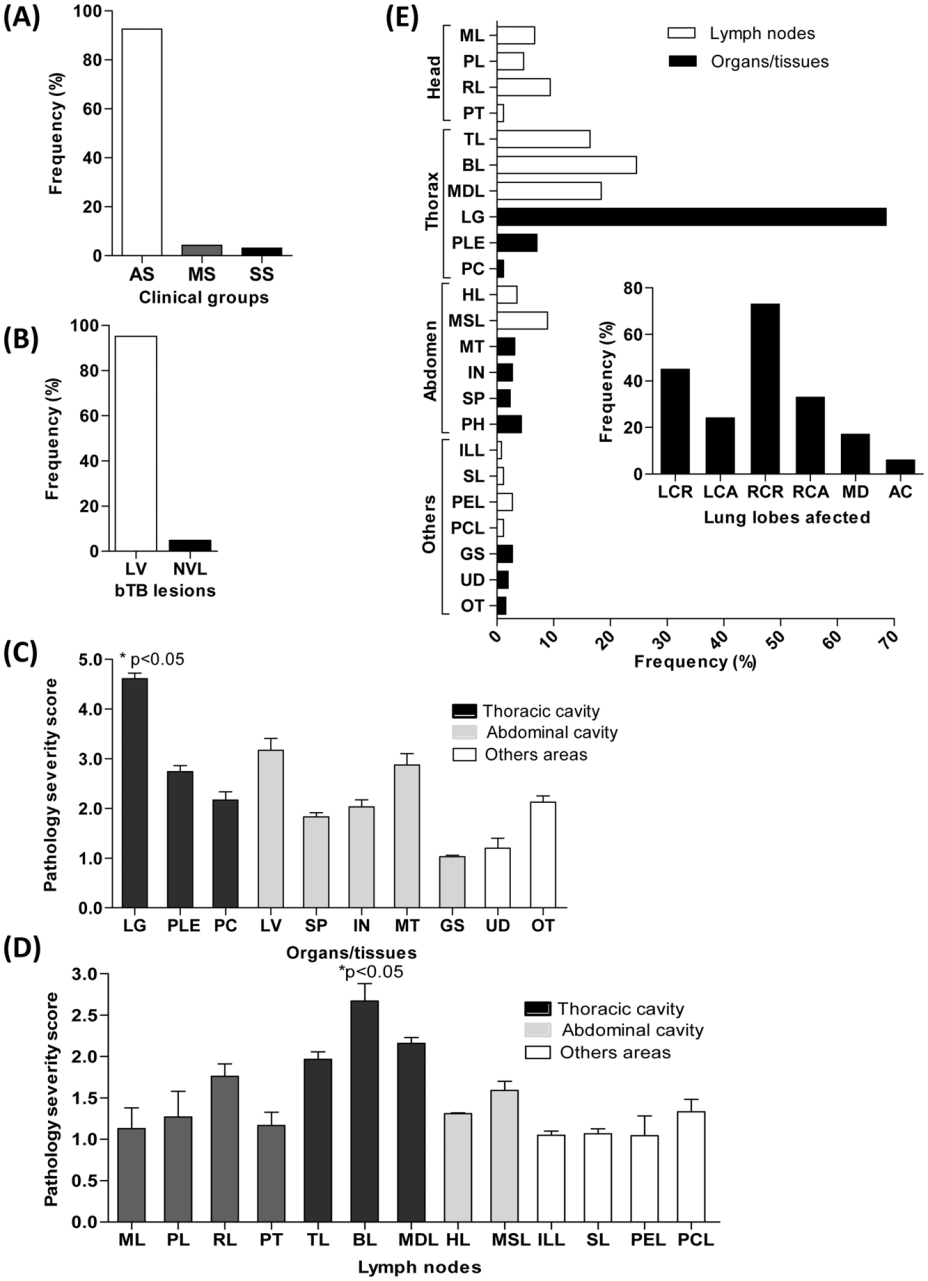
Clinical and gross pathology findings in cattle naturally infected with *M. bovis*. (**A**) Following clinical examination, animals bTB-positive (n = 247) were categorized according to their clinical status into asymptomatic (AS, n = 228), moderate symptoms (MS, n = 11), severe symptoms (SS, n = 8). (**B**) Gross pathology analysis further divided the PPD+ asymptomatic animals into two groups: the presence of visible bTB-lesions (VL) or absence of visible bTB-lesions (NVL). (**C and D**) Severity of tissue gross pathology in asymptomatic bTB bovines was scored by applying a previously described semi-quantitative scoring system (Vordermeier et al, 2002)**.** Results shown are median of scores ± SEM. (**C**) **Organs/Tissues:** thoracic organs and tissues (lung (LG), pleura (PLE), pericardia (PC)); abdominal (liver (LV), spleen (SP), intestine (IN), mesentery (MT), genitor-urinary system (GS), as well as udder (UD), other tissues (OT)); (**D**) **Lymph nodes:** head lymph nodes (mandibular (ML), parotid (PL), retropharyngeal (RL) lymph nodes and palatine tonsil (PT)); thoracic lymph nodes (tracheobronchial (TL), bronchial (BL) and mediastinal (MDL)); abdominal lymph nodes (hepatic (HL), mesenteric (MSL)) as well as Iliac (ILL), Sciatic (SL), pre-scapular (PEL) and pre-crural (PCL) lymph nodes. (**E**) Frequency of bTB-lesions in different organs/lymph nodes affected of asymptomatic bTB bovines. Legends as described in (**C**) and (**D**). (**E, inset**) Frequency of lung lobes affected (left cranial lobe (LCR), left caudal lobe (LCA), right cranial lobe (RCR), right caudal lobe (RCA), middle lobe (MD) and accessory lobe (AC) were determined.

Experimental and observational data have demonstrated that bovines display increased resistance to *M. bovis* infection [25], [31], [33]–[35]. Such resistance has been thought to impact surveillance programs as well as bacterial dissemination given a possible delay between bTB testing and cattle elimination. To gain insight on the immune-pathological responses induced during the window between infection and appearance of bTB-associated clinical signs, we have studied a cohort of 247 PPD-positive bovines naturally infected with *M. bovis* **(** **Figs. 1** **and** **2** **).** In this cohort, 92.3% of the animals (228 bovines) displayed no clinical signs suggestive of mycobacterial infection **(**asymptomatic group, AS; **Fig. 2A** **)**. At the post-mortem evaluation, 217 bovines (95.2% of the AS group) presented severe visible bTB-lesions **(** **Figs. 1A** **and** **2B** **)** with varying degrees of gross pathology scoring **(** **Figs. 2C and D** **)**. The majority of animals presented lesions in the lungs, primarily in right cranial lobe and in pulmonary-associated lymph nodes **(** **Fig. 2E** **and** ***inset*** **)**, suggesting the aerogenous route of transmission was probably the main via of infection in the studied bovine herds. Thus, these findings demonstrate different organs are affected by *M. bovis* and suggest that following primary infection, the bacterium can disseminate to a variety of tissues during natural infection.

**Figure 3:**
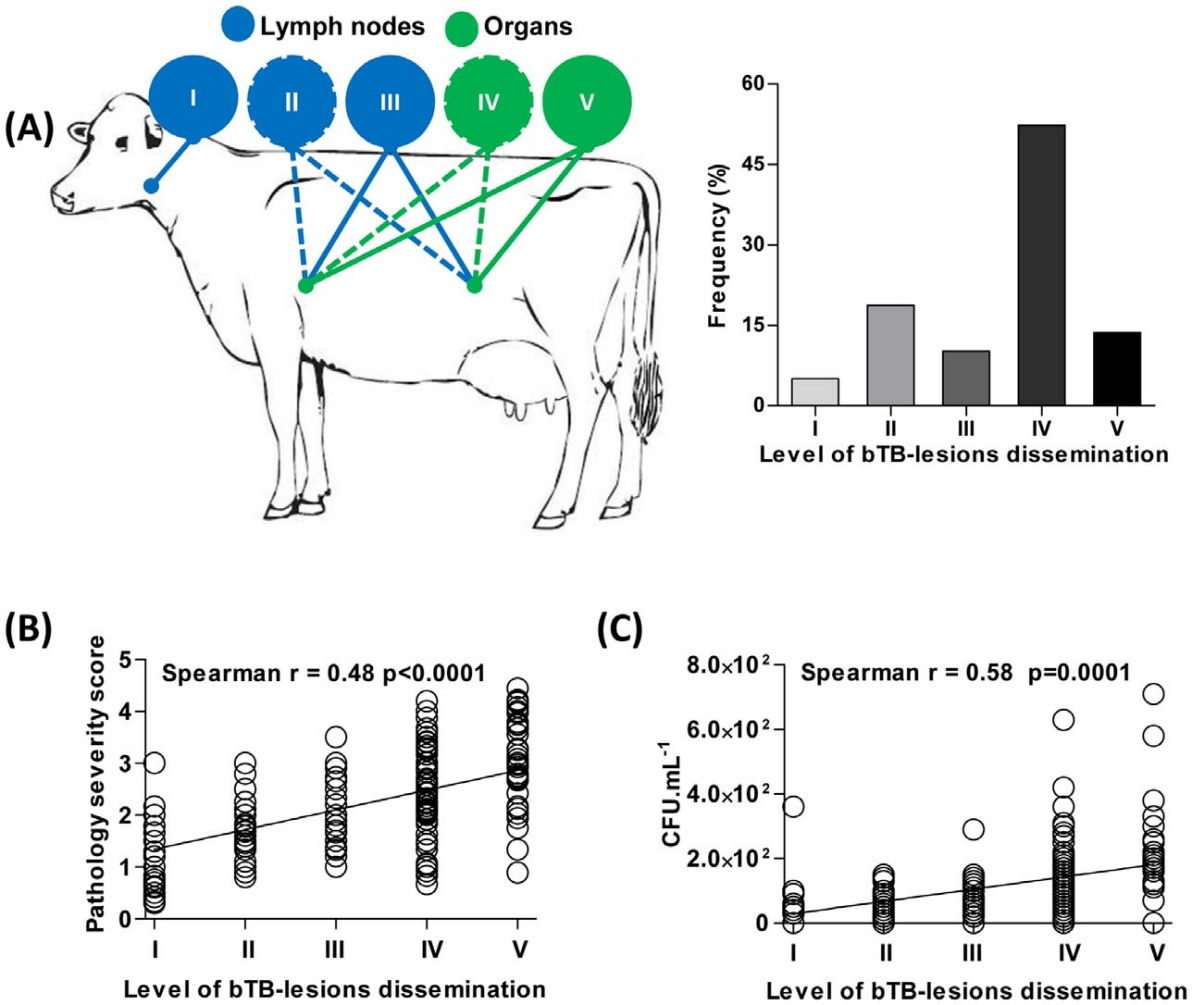
Anatomical dissemination of bTB-lesions and infection burden in asymptomatic cattle naturally infected with *M. bovis.* (**A**) Frequency of animals categorized according to their anatomical dissemination of bTB-lesions into five levels: I, lesions of bTB in the head lymph nodes, including retropharyngeal, mandibular, and parotid lymph nodes; II, presence of lesions of bTB in thoracic lymph nodes, including the mediastinal, bronchial, tracheobronchial lymph nodes, *or* in abdominal lymph nodes, including mesenteric, inguinal, gastric, hepatic, splenic, renal, sub-iliac, medial and lateral iliac lymph nodes; III, simultaneous presence of lesions suggestive of bTB in thoracic *and* abdominal lymph nodes; IV, presence of lesions of bTB in organs of the thoracic *or* abdominal cavity; and V, simultaneous presence of lesions of bTB in organs of thoracic *and* abdominal cavities. Schematic cartoon of the scoring system is represented. Continuous line represents both cavities affected (thoracic and abdominal); dotted line represents only one cavity affected. Green balloons indicate affected organs and blue balloons indicate affected lymph nodes. Correlation between levels of lesion dissemination and pathology severity (**B**) or mycobacterial loads (**C**) in cattle naturally infected with *M. bovis*. In (**B**), the results are expressed applying the gross pathology severity semi-quantitative scoring per animal previously described in [26], and (**C**) as number of CFU.mL^−1^ (colony-forming units per mL of granulomatous tissue homogenate). Spearman’s correlation indexes (Spearman’s r and p values) are shown in the graphs.

To study disease severity and possible immunopathological correlates of *M. bovis* infection, we developed a score system based on anatomical bTB-lesions dissemination **(** **Fig. 3A** **)**. Using this system, 66.2% (n = 151) of bovines were defined as levels IV and V **(** **Fig. 3A** **),** pointing out a possible connection between disseminated infection and disease activity. To validate this hypothesis, we then performed Spearman correlations between viable bacterial loads (CFU counting, **Fig. 1A**), pathology severity (PS score, **Fig. 1C and D**) and bTB lesion dissemination. As demonstrated in **figure 3** **(B** and **C)**, our score system positively correlates with gross pathology severity as well as tissue bacterial loads. These results formally demonstrate that naturally infected cattle with increased pathology severity display higher loads of viable *M. bovis*. These findings suggest asymptomatic animals could play an important role in bacterial transmission and maintenance of disease before diagnostic and elimination of bovine populations in nature.

### Granuloma Encapsulation Negatively Correlates with *M. bovis* Loads in Naturally Infected Cattle

**Figure 4:**
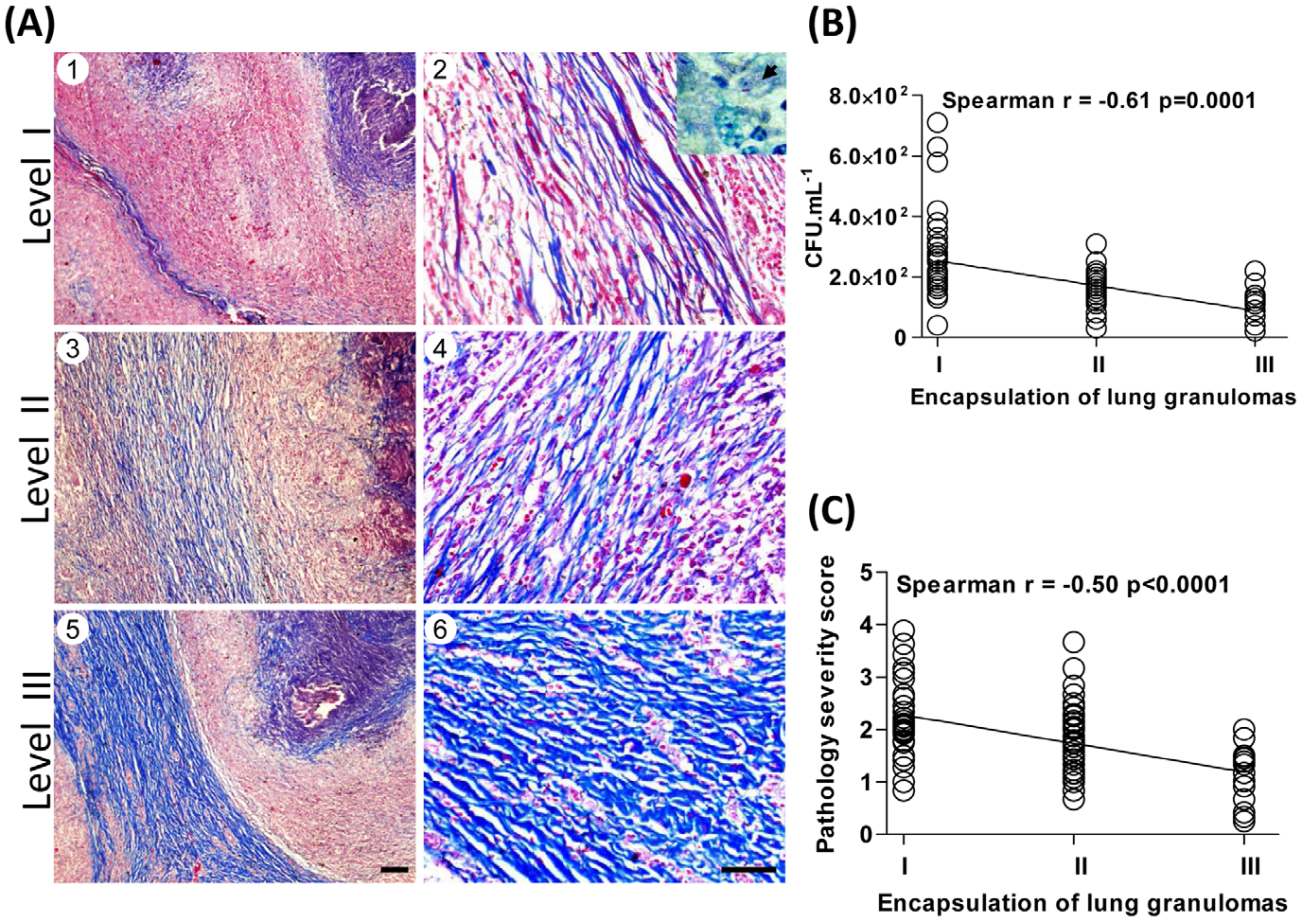
Histopathological analysis of granuloma encapsulation in asymptomatic cattle naturally infected with ***M. bovis.*** (**A**) Granulomas were formalin-fixed, paraffin-embedded and 4 µm-sections Massońs trichrome stained, categorized and scored according to their intensity of encapsulation of primary granulomas into three levels: (1 and 2) I, thin encapsulation; (3 and 4) II, dense fibrous capsule; and (5 and 6) III, thickly fibrous encapsulation. (A, inset) Acid Fast-Bacilli (AFB). (Left panels, slides shown at 10× magnification; Scale bar  = 100 µm. Right panels, slides shown at 40× magnification; Scale bar  = 50 µm. Inset, slides shown at 100× magnification. Correlation between intensity of granuloma encapsulation with mycobacterial loads (**B**) or gross pathology severity score (**C**) in cattle naturally infected with *M. bovis* are presented. Spearman’s correlation indexes (Spearman’s r and p values) are shown in the graphs.

The observed dissemination of *M. bovis* and the presence of severe bTB-lesions in asymptomatic animals suggest the existence of a robust immune response during natural infection [23]–[25], [34]. To investigate bovine-protective factors associated with control of natural infection, which could influence host disease-*M. bovis* interplay and transmission, we have performed a detailed study of the granuloma, a major structure known to be associated with containment of mycobacterial dissemination [14]–[16], 20. Following analysis of primary lesions from 217 infected AS animals (573 tuberculous granulomas), three major degrees (I-III) of encapsulation intensity were observed, **(** **Fig. 4A** **)** in which most of the animals (138; 64%) showed level III (thickly encapsulation), indicating an attempt to limit the infection in such studied cattle. Consistent with these results, a significant negative correlation between encapsulation and viable bacterial loads (Spearman r = −0.61, p = 0.0001) was observed **(** **Fig. 4B** **).** Moreover, gross pathology severity (Spearman r = −0.50, p<0.0001) **(** **Fig. 4C** **)** and AFB staining (Spearman r = −0.41, p<0.0001) (data not shown) were also found to negatively correlate with encapsulation**.** Interestingly, AFBs were found to be located mainly within the necrotic caseum centre of granulomas and rarely within macrophages, multinucleated giant cells or mineralized debris **(** **Fig. 4A** **,** ***inset*** **)**. These data suggest generation of granuloma encapsulation is important to contain *M. bovis* growth in naturally-infected bovine herds.

### Analysis of cellular profile of lung granulomatous response from bovines naturally infected with *M. bovis*

**Figure 5:**
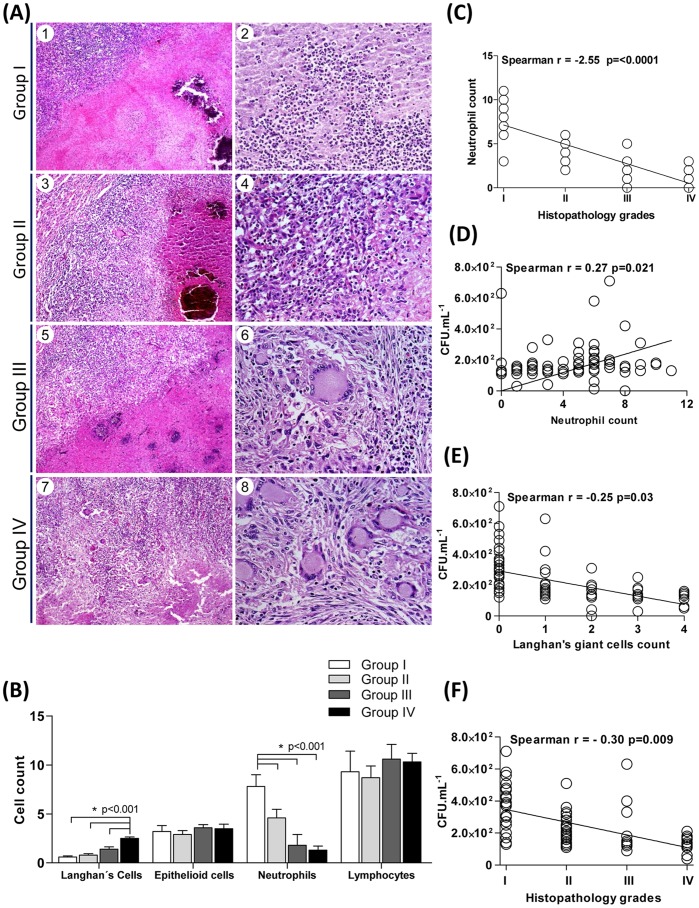
Histopathological analysis of cellular profile of granulomatous response in lung of asymptomatic cattle naturally infected with *M. bovis.* (**A**) Lung tissues were categorized according to the granuloma cellular response profile and tissue remodeling into four groups: I-IV. Representative lung-tuberculous granulomatous response patterns are shown: I (1 and 2) encapsulated granulomas with caseous necrosis areas and presence of several scattered lymphocytes and dense clusters of neutrophils near the capsule; II (3 and 4) encapsulated granuloma, with extensive areas of caseous necrosis. Granulomatous cellular response composed primarily of epithelioid macrophages, lymphocytes, multinucleated Langhańs giant cells and clusters of neutrophils; III (5 and 6) encapsulated granulomas, with extensive multicentric areas of caseous necrosis and centralized dystrophic mineralization. Granulomatous cellular response composed of epithelioid macrophages and scattered Langhan’s giant cells, which surround the necrotic areas with dense clusters of lymphocytes and few neutrophils; III (7 and 8) encapsulated granulomas, with extensive multicentric areas of caseous necrosis and centralized dystrophic mineralization. Granulomatous cellular response composed of epithelioid macrophages admixed with increased numbers of multinucleated Langhan’s giant cells, dense clusters lymphocytes and few neutrophils. Left panels, slides shown at 10× magnification; Scale bar  = 100 µm. Right panels, slides shown at 40× magnification; Scale bar  = 50 µm. (**B**) Results presented are mean ± SEM for each group shown in (**A**). Correlation between neutrophil counts and histopathology grades (**C**), Correlation between viable mycobacterial loads and neutrophils (**D**), multinucleated Langhańs giant cells counts (**E**) as well as lung-granulomatous response profile (**F**). Spearman’s correlation indexes (Spearman’s r and p values) are shown in the graphs (n = 168 animals for correlation figures).

We next performed a detailed analysis of the cellular profile of pulmonary granulomas of *M. bovis*-infected asymptomatic animals. As demonstrated in **figure 5A**, histological analysis of the lung tuberculous granulomatous response revealed four major histopathology groups (grades I-IV)**,** which differ on granuloma-associated cell type numbers: Langhańs multinucleated giant cells, epithelioid macrophages, neutrophils and lymphocytes **(** **Fig. 5B** **)**. As expected, multinucleated giant cells displayed a positive correlation with histopathology grades (Spearman r = 0.55, p = <0.0001). In contrast, neutrophil numbers presented a negative correlation with our defined histopathology grades (Spearman r = −2.55, p = <0.0001) (**Fig. 5C**). These data suggest lung granulomas from bovines naturally-infected with *M. bovis,* although chronically infected and encapsulated, are sites of bacterial growth that dynamically recruits neutrophils. In support of this hypothesis, we observed a positive correlation between neutrophil numbers and viable *M. bovis* (Spearman r = 0.27, p = 0.021) **(** **Fig. 5D** **).** In addition, *M. bovis* CFU counts negatively correlated with multinucleated giant cell numbers (Spearman r = −0.25, p = 0.03) **(** **Fig. 5E** **)** and lung histopathology grades (Spearman r = −0.30, p = 0.009) **(** **Fig. 5F** **)**. Together, these evidence suggest neutrophils and giant cells may play a role in regulating *M. bovis* growth in the lung during natural infection of bovines.

## Discussion

Although bTB is an important neglected zoonosis which significantly decreases livestock production and impacts public health, little is known about the dynamics of host-*M. bovis* interactions during natural infection. Moreover, the immunopathological parameters associated with protective host response in cattle during natural *M. bovis* infection remains largely unknown. In this study, we have performed a detailed analysis of important anti-mycobacteria host defense response components in a cohort of 247 naturally *M. bovis*-infected cattle. Despite the absence of clinical signs of bTB, the majority of infected cattle displayed high frequency and severity of the bTB-lesions in the lung (68.6%) (right cranial lobe) as well as pulmonary-associated lymph nodes. In addition, no correlation between the PPD size reactions and pathology severity was observed in our cohort (Spearman r = 0.01 p = 0.85).

Observations obtained from naturally *M. bovis*-infected cattle submitted to low-intensive farming conditions have demonstrated that the majority of lesions were present in mesenteric lymph nodes [32], [36]. In contrast, naturally *M. bovis*-infected bovines exposed to intensive husbandry systems display augmented frequency of bTB-lesions in the respiratory tract [31], [36], [37]. Thus, it is possible that intensive husbandry systems favor *M. bovis* dissemination among dairy herds as a result of increased animal contact [5], [38]. Consistent with this hypothesis, we have found that 66% of animals displayed pulmonary and systemic spreading of bTB infection (levels III/IV) **(** **Fig. 3A** **)** suggesting that exposure to the pathogen was first established in the lung tissue. Moreover, the high frequency of lesions observed in the respiratory tract suggests that the major route of *M. bovis* transmission was most likely aerogenous. These evidences are supported by previous studies which demonstrated that bovines infected via the intranasal route by *M. bovis* results in pathology confined to the respiratory tract [39]–[41]. Involvement of mesenteric lymph nodes was also observed in our cohort, although at a significantly lower frequency (8.8%) **(** **Fig. 2E** **)**, suggesting that infection by the oral route may occur simultaneously during natural infection.

It is commonly accepted that *M. bovis* primarily infects macrophages, where they are able to survive, replicate and disseminate into different anatomical sites [14], [15], [20]. Progression of mycobacterial disease and survival of the host are thought to depend on their ability to limit mycobacterial growth by an effective granulomatous response [14]–[16], [20], [42], [43]. In the case of experimental *M. bovis* infection in cattle, different stages of granuloma development have been observed to be associated with disease progression [19], [21], pointing out a dynamic process of the tuberculous granuloma structure. Our results confirm and extend previous studies [19], [21], which suggest an important role for the host granulomatous responses against *M. bovis* during natural infection. We found that anatomical dissemination of bacteria/lesions is associated with tissue mycobacterial loads as well as severity of the gross pathology, suggesting the existence of a dynamic host immune response during natural infection. Although further studies are needed to better characterize the process of *M. bovis* dissemination in naturally-infected bovines, the parameters presented here could be employed as predictive biomarkers of disease progression and utilized in control surveillance programs.

Cattle immune responses against *M. bovis* may be a result of several factors, such as strain resistance, infection route and encapsulation of the tuberculous lesions. Connective tissue deposition (encapsulation) is thought to limit dissemination of bacteria and play a critical role in controlling mycobacterial proliferation by entrapping bacilli inside the lesions [14], [20], [22], [25]. However, Liebana et al. have reported the absence of correlation between AFB numbers and stage of granuloma development during natural infection with *M. bovis* in England and Wales [31]. In our study, histological analysis of granulomatous response and tissue remodeling revealed high frequency of chronic lesions in different tissues, which negatively correlated with viable mycobacterial counts **(** **Figs. 4** **and** **5** **)**.

In order to better investigate the lesion development in cattle naturally infected with *M. bovis*, we first applied the methodology described by Wangoo et al. [21], which performed a descriptive study of the granulomatous responses in lymph nodes from cattle infected with *M. bovis* by the intratracheal route. The employment of such methodology in our samples led us to classify the majority of lesions in different organs/tissues (70–100%) as in the final stage of granuloma development, i.e. stage IV **(** **Table 1** **).** Due to increased resistance of cattle to *M. bovis* infection [2], [28]–[30], it is possible that during the natural infection, most of bTB-lesions found in asymptomatic animals are in advanced/chronic stage of development. Interestingly, we found that, despite the observed chronic stage of lesions, thickness of encapsulation could be employed as a marker of lesion development and allowed us to further classify the granulomas into three major groups (I-III) **(** **Fig. 4** **)**. The amount of connective tissue surrounding the granuloma (thin encapsulation - thickly fibrous encapsulation) negatively correlated with viable *M. bovis* or AFB staining, suggesting a pivotal role of granuloma encapsulation as a host response controlling mycobacterial proliferation during natural infection. In contrast, lymph nodes from experimentally *M. bovis*-infected cattle were found to present increased AFB numbers in advanced-stage granulomas [19], [21]. These data are in direct contrast with the findings obtained herein and could be explained by the employment of different models of infections, i.e. experimental vs natural infection. In addition, we have performed analysis of viable bacteria, which in comparison with AFB staining, more closely reflects the *M. bovis* loads present in the bTB lesions. The encapsulation response possibly may be induced by the bacillary burden in the granulomas. Nevertheless, animals with increased numbers of thickly encapsulated lesions were found to display lower bTB-lesions dissemination, suggesting induction of a mature connective tissue in the granulomas actively participates of anti-*M. bovis* immune responses during natural infection.

**Table 1:** Distribution and stage of histological development of primary tuberculous granulomas in cattle naturally infected with *Mycobacterium bovis.*

Distribution of granulomas		*Development stages of granulomas (%)*			
		I	II	III	IV
***Major organs/tissues***					
Thorax	Lung	1.9 (3/159)	1.2 (2/159)	11.3 (13/159)	85.5 (136/159)
	Pleura	0	22.2 (4/18)	5.6 (1/18)	72.2 (13/18)
	Hearth		0	0	0
	Pericardia	0	0	0	100 (6/6)
Abdominal	Liver	0	12.5 (1/8)	0	87.5 (7/8)
	Spleen	0	0	16.7 (1/6)	83.3 (5/6)
	Intestine	0	0	28.6 (2/7)	71.4 (5/7)
	Mesentery	0	0	25 (2/8)	75 (6/8)
	Genito-urinary system	0	28.6 (2/7)	0	71.4 (5/7)
Carcass	Udder	0	0	0	100 (5/5)
	Other tissues	25 (1/4)	75 (3/4)	0	0
***Lymph nodes***					
Head	Parotid	0	16.7 (2/12)	0	83.3 (10/12)
	Retropharyngeal	4.2 (1/24)	0	16.7 (4/24)	79.2 (19/24)
	Mandibular	0	5.9 (1/17)	52.9 (9/17)	41.2 (7/17)
	Palatine tonsil	0	0	0	100 (3/3)
Thorax	Tracheobronchial	2.4 (1/42)	4.7 (2/42)	14.3 (6/42)	85.7 (36/42)
	Bronchial	1.6 (1/63)	3.2 (2/63)	4.7 (3/63)	90.5 (57/63)
	Mediastinal	4.3 (2/47)	2.1 (1/47)	4.3 (2/47)	89.4 (42/47)
Abdominal	Hepatic	0	0	22.2 (2/9)	77.8 (7/9)
	Mesenteric	0	9.1 (3/33)	12.1 (4/33)	69.7 (23/33)
Others	Iliac	0	0	0	100 (2/2)
	Sciatic	0	0	0	100 (3/3)
	Prescapular	0	14.3 (1/7)	0	85.7 (6/7)
	Precrural	0	0	0	100 (3/3)

The majority of pulmonary granulomas investigated in our cattle cohort presented as encapsulated lesions with multiple intragranulomatous areas of caseous necrosis and the presence of dystrophic mineralization, which according to the criteria established for granuloma in lymphoid tissue during experimental infection, can be classified as chronic bTB-lesions (stage III/IV) [19], [21]. Furthermore, histomorphological analysis of the lung granulomas revealed major differences in cell type counts **(** **Fig. 5** **).** Consistent with these results, the process of granuloma maturation involves the migration of phagocytes and lymphocytes to the inflammation site in response to persistent mycobacterial stimuli [15], [16], [20], [22], [42]. An effective anti-mycobacteria host response primarily rely on cell-mediated immune response, controlled by cytokines such as IFN-γ produced by antigen-specific T cells [17], [24], [26], [44], [45]. Although the protective role of cell-mediated immune responses is unknown in cattle naturally infected with *M. bovis*, in the present study, we have observed significant correlations between neutrophil or Langhan’s giant cell numbers and granuloma mycobacterial loads. Neutrophilic infiltrate was observed particularly in early stages of granuloma infection [19], [21] and could be important for granuloma formation. Also, neutrophils have been suggested to play a regulatory anti-mycobacterial role [46], [47]. We have a found a positive correlation between neutrophil numbers and CFU counts, suggesting that bacillary burden induce neutrophil recruitment and/or maintenance into the granulomas. In contrast, neutrophil could potentially play a detrimental role by favoring mycobacterial growth in granulomas during natural infection [48], [49]. A negative correlation between multinucleated giant cell numbers and *M. bovis* CFU counts in granulomas suggests, as expected, that activated multinucleated macrophages contribute to the control of this important bovine pathogen. Data from experimental models have demonstrated Langhan’s-type multinucleated giant cells can be found in all stages of development of lymph node granulomas [19], [21]. Together, our findings indicate that *M. bovis*-induced granulomas in the lungs are dynamic lesions in which the cell populations change over the course of disease, stimulating a diverse milieu during infection. The physiopathology of this complex structure during natural infection of *M. bovis* merits further investigation.

Cattle are natural hosts of *M. bovis*, which besides being an economically important pathogen for international trade, is an imminent risk to public health. The evidence presented in this study could reflect a situation of bTB found in Brazil, which may not be transferable to other countries. Nevertheless, the data a presented here offer basic information on the host response during the natural infection with *M. bovis,* which could be utilized as a potential source for biomarkers to test novel vaccine/adjuvant molecule candidates as well as efficient diagnostic methods. In addition, our findings may be important to reveal new components to understand the immunopathogenesis of the bTB and contribute to the establishment of rational strategies for bTB infection surveillance and control.

## Materials and Methods

### Animals and Ante-mortem Evaluation

The study obtained ethical clearance from the Universidade do Estado de Santa Catarina ethical review committee (P#1.13.10). Federal government inspection abattoirs comply with PNCETB 06/2004 and MAPA 03/2000, which follow International Ethical Guidelines of Animal Welfare. The study population was comprised of 247 crossbred Holstein/Jersey cows between the ages of ∼1.6 to 11 years which were mandatorily conducted to abattoirs after a positive reaction for the single intradermal comparative cervical tuberculin test (SICTT; PPD) following Brazilian regulations [50]. These animals derived from 18 dairy farms with intensive husbandry systems, which experienced 23 bTB outbreaks between 2009 and 2011. Farms were located in Santa Catarina State, Brazil, and maintained under surveillance control against bTB following Brazilian’s regulations. SICTT tests were performed in accordance with regulations set forth by the Brazilian Department of Agriculture (MAPA) [50]. Briefly, two sites located 12 cm to 15 cm apart on the cervical area of the mid-neck were shaved and skin thickness was measured using calipers. The first site was injected with 0.1 mL of bovine PPD (PPD-B - *M. bovis* strain AN5, 1 mg protein/mL), while the second site was injected with 0.1 mL of avian PPD (PPD-A - *Mycobacterium avium* strain D4, 0.5 mg protein/mL) [50]. After 72 hours, skin thickness at the injection sites was measured, and the difference between the reaction sizes for the two injection sites was determined. An animal was classified as PPD-positive if the skin thickness at the PPD-B injection site was at least 4 mm greater than the skin thickness at the PPD-A injection site [9].

During ante-mortem analysis, animals were classified into the following three clinical stages based on symptoms observed during clinical evaluation: ***absence,*** absence of clinical signs; ***moderate,*** weight loss, hyporexia, coughing intermittently; and ***severe,*** extreme weight loss, weakness, hyporexia, hemoptysis, dyspneia, progressive cough and tuberculous mastitis.

### Post-mortem Examination and Pathology Analysis

All major body organs and lymph nodes were examined for the presence of visible lesions suggestive of bTB disease. Organs and lymph nodes were cross-sectioned in 0.5 cm to 1 cm intervals and examined individually for the presence of lesions. Organ and tissue samples from animals with or without bTB visible lesions (VL) were collected for *M. bovis* culture and PCR analysis as well as for histopathological examination. Only animals displaying sample tissues positive for *M. bovis* by culture, PCR or direct examination *(*Ziehl*-*Neelsen – acid fast bacilli - staining) were included in the study. The anatomical dissemination of the visible gross pathological lesions in different organs and tissues were scored according to the following system: I = presence of bTB-lesions in the lymph nodes of the head, including the left and right medial and lateral retropharyngeal, left and right mandibular, and left and right parotid lymph nodes; II = presence of bTB-lesions in thoracic lymph nodes, including the cranial and caudal mediastinal, cranial tracheobronchial, left and right tracheobronchial lymph nodes, *or* in abdominal lymph nodes, including mesenteric, deep and superficial inguinal, gastric, hepatic, splenic, renal, subiliac, medial and lateral iliac lymph nodes; III = simultaneous presence of bTB-lesions in thoracic *and* abdominal lymph nodes, including lymph nodes already mentioned above; IV = presence of bTB-lesions in organs of the thoracic *or* abdominal cavity, with or without the presence of lesions in the draining lymph nodes associated with the organ; and V = simultaneous presence of bTB-lesions in organs of thoracic *and* abdominal cavities, with or without the presence of lesions in the draining lymph nodes associated with the organ. Out of the cattle with score IV or V, most of them (85%) displayed also lesions in draining lymph nodes.

The severity of the visible gross pathological changes in the major body organs and lymph nodes were classified by applying the semi-quantitative scoring of gross lesions previously described by Vordermeier et al. [26]. Briefly, each lung lobe, including left cranial, left caudal, right cranial, right caudal/middle, and accessory lobes, was cross-sectioned at 0.5 to 1.0 cm intervals and scored from 0 to 5 depending on the number of lesions and extent of pathology observed, 0 being no visible lesions and 5 being coalescing gross lesions. The scores of the individual lobes were summed to calculate the lung score. The major **organs/tissues,** including the pleura, pericardia, liver, spleen, intestine, mesentery, uterus, ovaries, kidney, bladder and muscular tissue, were scored as well. The **lymph nodes**, including the mandibular, parotid, medial retropharyngeal, palatine tonsil, bronchial, mediastinal and tracheobronchial, hepatic, mesenteric, iliac, sciatic, pre-scapular and pre-crural lymph nodes, were cross-sectioned at 0.5 cm intervals and were scored using a score of 0 to 3, 0 being no visible lesions and 3 being extensive or coalescing gross lesions. Pathology scores were combined to determine mean gross pathology severity score per animal.

### Bacteriology and Molecular Typing of *M. bovis* from bTB Lesions

Tissue sections collected at post-mortem from lymph node and lung samples were individually homogenized using a rotating-blade macerator system (Tissue ruptor®). One milliliter of homogenate was decontaminated and concentrated by Petroff’s Sodium Hydroxide method according to [9]. Culture and enumeration of bacteria (CFU.mL^−1^ tissue homogenate) was performed by inoculating 100 µL of decontaminated tissue homogenate in Ogawa-Kudoh (OK) agar containing sodium pyruvate (12 mg/mL) and counting colonies after aerobic incubation at 37°C for 8 weeks. After growth in OK+pyruvate, colonies were molecularly typed by PCR. Briefly, purified mycobacterial DNA from colonies extracted as previously described [51] was used as template for PCR amplification of the following multi-copy insertion gene IS1081 (∼135 bp), present in the *M. tuberculosis* complex organisms (Fig. 1B) [52] and RvD1Rv2031c (∼500 bp) a polymorphic region of 2900 bp in the *M. bovis* genome which was not homologous in the genomes of *M. tuberculosis* and *M. avium* (Fig. 1C) [53].”

### Histopathological Analysis

Tissues samples were fixed in 10% neutral buffered formalin and dehydrated in graded ethanol solutions. After dehydration, samples were paraffin-embedded, sectioned (4 µm), and stained by hematoxylin and eosin (H&E), Massońs trichrome or AFB method. Microscopically, the granulomas from **lymph nodes** and major **organs/tissues** (pericardia, pleura, liver, spleen, intestine, mesentery, uterus, ovaries, kidney, bladder, adrenal and muscular tissue) were classified into the following four categories [21] according to the development of the lesion: stage I - initial or early lesions; stage II - solid granulomas; stage III - minimal necrosis; or stage IV - necrosis and mineralization. Additionally, three levels of granuloma encapsulation were identified following classification being proposed in this study: level I - thin encapsulation; level II - dense fibrous capsule; level III - thickly fibrous encapsulation. All **lung granulomas** were subjected to systemic histopathological examination. Specifically for the classification of lung granulomas, the following proposed criteria were used to classify in four different groups of lesions: **group I (score  = 1)** granulomas circumscribed by fibrous encapsulation with caseous necrosis areas and presence of several scattered lymphocytes and dense clusters of neutrophils near the capsule. Epithelioid macrophages and low Langhańs giant cell count, which surround the necrotic areas; **group II (score  = 2)** – granuloma circumscribe by fibrous encapsulation, with extensive areas of caseous necrosis. Granulomatous cellular response composed primarily of epithelioid macrophages, lymphocytes, moderate Langhańs giant cell count and clusters of neutrophils, which surround the necrotic areas and extend until capsule; **group III (score  = 3)** – granulomas circumscribed by fibrous encapsulation, with extensive multicentric areas of caseous necrosis and centralized dystrophic mineralization. Granulomatous cellular response composed of epithelioid macrophages and moderate amount of scattered Langhan’s giant cells, which surround the necrotic areas with dense clusters of lymphocytes and few or absent clustered neutrophils near the fibrous capsule; **group IV (score  = 4)** – granulomas encapsulated, with extensive multicentric areas of caseous necrosis and centralized dystrophic mineralization. Granulomatous cellular response composed of epithelioid macrophages admixed with large amount of Langhańs giant cells, which surround the necrotic areas with dense clusters lymphocytes and few or absent neutrophils near the fibrous capsule. For the histopathological analysis, total number of granulomatous cellular response cells was counted in 10 microscope fields (1×100 magnification) per lung granuloma section. Mean and standard deviation were determined for each group. For coalescing or multicentric lesions in the histomorphological analysis, the primary lesion or more chronic/advanced lesion/stage was evaluated. The relative number of acid-fast bacilli (AFB) found in each ZN-stained section was estimated as well.

### Statistical Analysis

Statistical analysis was carried out using GraphPad Prism 5 (GraphPad Software Inc., San Diego, CA, USA). Correlations between bacterial load, level of granuloma-encapsulation, histopathological lung-granulomatous response, gross pathology, and lesion distribution were assessed by nonparametrical analysis applying the Spearman rank correlation. Spearman’s correlation coefficients (r_s_) and p-values are provided.
